# Un myxome poly-embolique de l'oreillette gauche

**DOI:** 10.11604/pamj.2015.20.336.6562

**Published:** 2015-04-08

**Authors:** Nabil Elmalki Berrada, Iliyasse Asfalou, Maha Raissouni, Younes Moutakiallah, Aatif Benyass

**Affiliations:** 1 Service de Cardiologie, Hôpital Militaire d'Instruction Mohammed V, Rabat, Maroc; 2 Service de Chirurgie Cardiovasculaire, Hôpital Militaire d'Instruction Mohammed V, Rabat, Maroc

**Keywords:** Myxome, embolie, Accident vasculaire cerebral, infarctus, Myxoma, embolism, Cerebrovascular accident, infarction

## Abstract

Le myxome cardiaque est une tumeur histologiquement bénigne, souvent découverte fortuitement à l’échocardiographie. Elle reste néanmoins grave par ses complications, notamment emboliques. Nous rapportons le cas d'un myxome de l'oreillette gauche compliqué de syndrome coronarien aigu et de multiples accidents vasculaires cérébraux ischémiques. Le diagnostic d'accidents emboliques est retenu devant l'association d'une tumeur intracardiaque emboligène, des AVCI et un SCA en l'absence de lésions d'athérosclérose et l'absence de thrombus intracardiaque. Le patient a bénéficié d'une exérèse chirurgicale de la tumeur avec des suites opératoires simples. Nous illustrons à travers cette observation le potentiel embolique du myxome qui rend cette tumeur redoutable et nous insistons sur l'intérêt d'une prise en charge chirurgicale urgente.

## Introduction

Bien que le myxome soit la tumeur cardiaque primitive la plus fréquente chez l'adulte, il ne représente que 0,25% des cardiopathies. Il s'agit d'une tumeur histologiquement bénigne, souvent découverte fortuitement à l’échocardiographie. Elle reste néanmoins grave par ses complications, notamment emboliques.

## Patient et observation

Monsieur F. M, âgé de 75 ans, hypertendu et ancien tabagique, est admis pour un syndrome coronarien aigu sans sus décalage du segment ST à haut risque (douleur thoracique angineuse remontant à deux jours avec un ECG normal à l'admission et une troponine élevée à 40x la normale. L'anamnèse trouve une notion d'accident ischémique transitoire survenu il y a deux ans, négligé par le patient.

**Figure 1:**
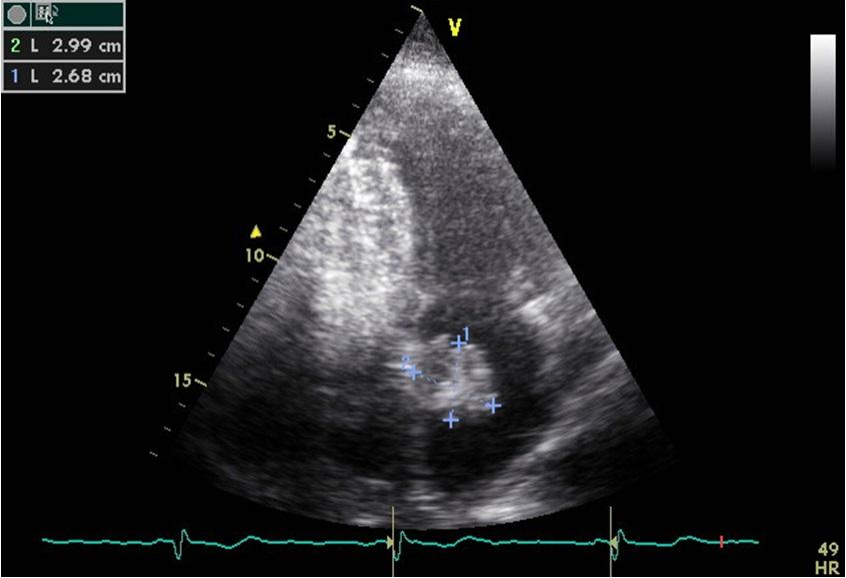
Myxome de l'oreillette gauche à l’échocardiographie transthoracique

**Figure 2:**
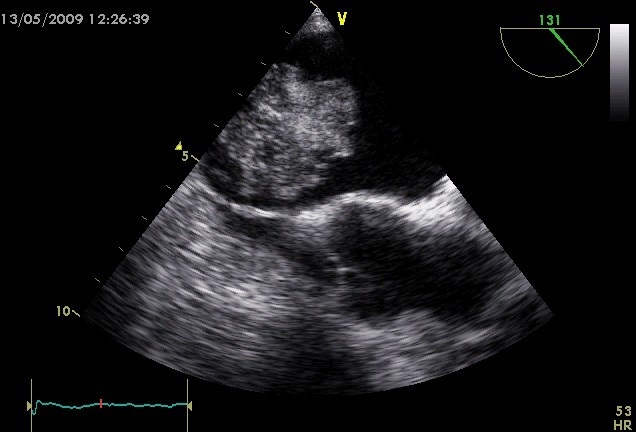
Myxome de l'oreillette gauche à l’échocardiographie transoesophagienne

L’échocardiographie transthoracique puis transoesophagienne montrent un VG hypertrophique, non dilaté, de contractilité normale et de fonction systolique conservée avec présence au niveau de l'oreillette gauche d'une masse polypoïde, de contours irréguliers, très mobile insérée sur le septum inter-auriculaire mesurant 34mmx 27mm. Cette masse évoque en premier un myxome de l'OG (Figure 1, Figure 2). Le patient est mis sous un traitement médical associant Aspirine, Clopidogrel, Enoxaparine, Atenolol et Simvastatine. La coronarographie montre des coronaires angiographiquement normales avec un trajet intra-myocardique de l'artère interventriculaire antérieure. L’évolution est marquée par la survenue d'une hémiplégie droite avec participation faciale.

**Figure 3:**
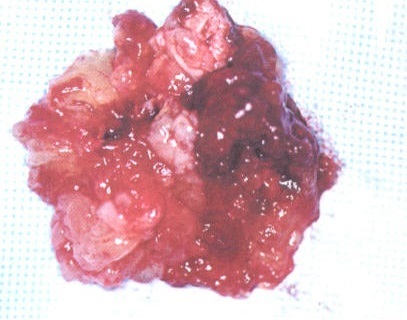
Myxome polypoïde et friable, pièce opératoire

La TDM et l'IRM cérébrales montrent un accident vasculaire cérébral ischémique (AVCI) sylvien gauche avec de multiples foyers d'AVCI anciens. L’écho-Doppler artériel des troncs supra-aortiques objective une surcharge athéromateuse sans sténose significative. Le patient fait l'objet d'une exérèse de la tumeur sous circulation extracorporelle avec des suites opératoires simples. L'examen anatomopathologique de la pièce opératoire confirme la nature myxomateuse de la tumeur (Figure 3).

## Discussion

Le myxome est la tumeur cardiaque la plus fréquente, représentant 50% des tumeurs cardiaques primitives [1]. Le myxome est histologiquement bénin certes, mais constitue toutefois, une tumeur redoutable par ses complications lourdes. Parmi celles-ci les complications emboliques sont bien connues et surviennent dans 45 à 60% des myxomes du coeur gauche et peuvent concerner les différents organes (arbre vasculaire cérébral, membres, rein, rate, coronaires, aorte abdominale) [2–4].

Les embolies artérielles à partir de myxome concernent le réseau coronaire dans 10% des cas [5, 6] et le réseau artériel cérébral dans 50% des cas [7]. Les emboles correspondent à des fragments de la tumeur ou à des thrombi formés à sa surface. L’échocardiographie est l'examen clé du diagnostic positif, avec une sensibilité de 93% pour la voie trans-thoracique et de 97% pour la voie trans-œsophagienne [3].

L’échocardiographie permet aussi de prédire le risque embolique du myxome. En effet, la morphologie du myxome est corrélée au risque embolique. Ainsi, les tumeurs villeuses et polypoïdes sont plus fragiles et embolisent plus souvent que celles à surface lisse et régulière [8]. La taille du myxome qui peut aller de quelques millimètres à plus de 15cm [9] n'est pas corrélée au risque embolique [8].

Le traitement du myxome est la résection chirurgicale sous circulation extracorporelle, avec un risque de récidive tardive chiffré à 2% [10]. Notre patient porteur d'un myxome de l'oreillette gauche avec des caractères morphologiques prédictifs d'un grand risque embolique, présente un syndrome coronarien aigu sans sus décalage du segment ST (NSTEMI) à coronaires angiographiquement normales et des AVCI multiples. Il s'agit vraisemblablement dans ce contexte d'accidents emboliques coronaire et cérébrale compliquant le myxome de l'OG.

Le diagnostic d'accidents emboliques est retenu devant l'association d'une tumeur intracardiaque emboligène, des AVCI et un syndrome coronarien aigu en l'absence de lésions d'athérosclérose (coronarographie normale, écho-Doppler artériel des troncs supra-aortiques normal) et absence de thrombus intracardiaque.

## Conclusion

Les accidents emboliques constituent une des complications révélatrices des myxomes, qui assombrissent le pronostic de ces tumeurs bénignes. La prise en charge chirurgicale est urgente notamment en présence de caractères morphologiques prédictifs d'embolie à l’échocardiographie.
